# Pediatric venous thromboembolism: incidence and patient profile in a single Brazilian institution

**DOI:** 10.1016/j.htct.2024.06.006

**Published:** 2024-09-07

**Authors:** Liana Ariel de Siqueira Lira, Jorge David Aivazoglou Carneiro, Maria do Carmo Menezes Bezerra Duarte

**Affiliations:** a Instituto de Medicina Integral Prof. Fernando Figueira (IMIP), Recife, Pernambuco, Brazil; b Instituto da Criança, Hospital das Clínicas da Faculdade de Medicina da Universidade de São Paulo (FMUSP), São Paulo, SP, Brazil

**Keywords:** Thrombosis, Venous thromboembolism, Child, Adolescent

## Abstract

**Background:**

As the diagnosis of Pediatric venous thromboembolism has dramatically increased in recent decades, this study aims to evaluate these patients, determining the incidence and describing their biological and clinical characteristics.

**Methods:**

An observational, cross-sectional study was conducted at a Brazilian quaternary hospital between January 2022 and February 2023. Under 18-year-old hospitalized patients with a confirmed diagnosis of venous thromboembolism were included, while those with arterial or chronic thrombosis were excluded. Data on biological and clinical characteristics, diagnosis and treatment were evaluated. A descriptive data analysis was performed and the incidence of hospital-associated thrombosis was calculated.

**Results:**

Thirty-nine pediatric patients were evaluated. The incidence of hospital-associated thrombosis was 19.9 cases per 10,000 pediatric hospitalizations. Median age at diagnosis was four months (range: 12 days-17 years). Most of the patients (66.7%) were asymptomatic, with venous thromboembolism being diagnosed incidentally. In all cases, at least one risk factor was identified and in 74.6% of cases four or more factors were present. The principal risk factors were the presence of a central venous catheter (89.7%) and infection (89.7%). Thrombogenic comorbidities, particularly congenital heart disease, were present in 48.7% of patients.

**Conclusions:**

The incidence of venous thromboembolism found in the present study was lower than rates reported in developed countries. The principal characteristics of this sample were a greater frequency of central venous catheter and infection as risk factors, and the fact that the cases consisted mainly of newborns and individuals with heart disease.

## Introduction

Venous thromboembolism (VTE) encompasses deep vein thrombosis (DVT) and pulmonary embolism (PE). The first reports on the incidence of VTE in the pediatric population are from Canadian records of the 1990s, with a reported annual rate of 0.07 cases per 10,000 children/adolescents.^1^ Studies conducted over the past two decades have reported an incidence of 0.14-0.49 cases/10,000 children/adolescents.^2^, ^3^, ^4^

The rise in the incidence of VTE in the pediatric population is even more significant among hospitalized children, having increased from 5.3 cases/10,000 pediatric hospital admissions in the 1990s to 58/10,000 in 2007 and 106/10,000 in 2019.^1^^,^^5^^,^^6^ This rise has been attributed both to an increase in suspected diagnosis and an actual increase in the number of cases, explained by the longer survival of critically ill children/adolescents and an increase in central venous catheter (CVC) use.^7^^,^^8^

Two incidence peaks have been reported: children <12 months old (9-30% of cases), particularly newborns, and adolescents 11-18 years old (30-54%).^1^^,^^2^^,^^6^^,^^9^ Risk factors are present in almost all pediatric patients with VTE with only 2-8.5% of cases being idiopathic.^2^^,^^9^ The most commonly reported risk factors are the presence of CVC (most closely associated with VTE), infection, malignancy, nephrotic syndrome, obesity and previous surgery.

Identifying the risk factors constitutes the main strategy for reducing pediatric VTE.^10^^,^^11^ Consequently, risk stratification scores for the pediatric population has become an important objective over the past decade.^12^, ^13^, ^14^, ^15^ However, the difficulty in establishing these scores lies in differences encountered in the profiles of hospitalized patients, in the levels of complexity of the hospitals analyzed, and in study designs.^11^

Almost all the data on VTE in pediatric patients originate from developed countries, specifically the United States, Canada and European countries. Two recent studies conducted in developing countries (India and Pakistan), identified different VTE characteristics, particularly regarding age, certain risk factors and incidence.^16^^,^^17^

The effect of genetic differences and demographic aspects on the epidemiology of VTE in adults has already been demonstrated.^18^ However, the epidemiology and local variations in VTE in the Brazilian pediatric population remain unknown. Multicenter studies are difficult to conduct due to the need for qualified laboratories, the sheer size of the country, the low incidence of the disease, and the lack of specialists in pediatric hemostasis.^19^ Consequently, this study aimed to evaluate the frequency of VTE and the characteristics of pediatric patients with VTE in a Brazilian quaternary hospital.

## Methods

### Study design and population

This observational, cross-sectional, single-center study, conducted between January 2022 and February 2023, included under 18-year-old hospitalized patients with an imaging-confirmed diagnosis of VTE. Patients with arterial thrombosis or chronic thrombosis were excluded. Chronic thrombosis was defined according to the signs and symptoms or thrombus characteristics on imaging exams, such as calcification or venous compressibility.^20^ Clinically unsuspected VTE was defined as VTE diagnosed by imaging test performed to screen for thrombosis or as an incidental finding (test requested for another reason) in the absence of any sign or symptom associated with VTE.^20^

The study center, a quaternary referral hospital that operates exclusively within the public healthcare system (Sistema Único de Saúde - SUS), receives an average of 15,000 pediatric hospitalizations annually. Its pediatric facilities include a neonatal intensive care unit (ICU), and general, cardiology, oncology and surgical wards and ICUs, thus demonstrating the diverse groups at risk of VTE. In recent years, a pediatric hematology unit for inpatients and outpatients was established.

### Ethical aspects

The institution's internal review board approved the protocol of this study under reference number 5.371.804 (CAEE: 55563422.9.0000.520).

### Data collection

An active search for potential participants was performed by screening for hospitalized pediatric patients with VTE using three different mechanisms: contacting the hospital radiologist with respect to cases of imaging-diagnosed VTE, the pediatrician for confirmed cases of VTE, and the pharmacist for patients taking parenteral anticoagulants. The medical records of the patients identified were verified to determine eligibility. Data were collected retrospectively from the medical charts and a specific questionnaire was used to interview the patients and their parents or guardians.

The variables evaluated were: age; sex; ethnicity; signs and symptoms of VTE; risk factors for VTE; duration of hospitalization prior to the diagnosis of VTE; need for assisted mechanical ventilation (AMV); days of AMV use until the diagnosis of VTE; diagnosis imaging modality: Doppler ultrasonography, echocardiogram, computed tomography (CT) with contrast, CT angiography, magnetic resonance angiography; location of VTE: intracardiac (right atrium), cervical, lower limb, upper limb, cerebral sites, pulmonary embolism, others; treatment provided: anticoagulant drugs, thrombolysis, thrombectomy; and outcome: discharge from hospital or death.

Based on the International Society on Thrombosis and Hemostasis (ISTH) recommendations for standardized risk factor definitions in pediatric hospital-acquired VTE,^21^ the following risk factors were evaluated: (a) Thrombogenic comorbidities: nephrotic syndrome, autoimmune/inflammatory disease, congenital heart disease, cancer, and obesity - evaluated in children >2 years old as body mass index (BMI) above the 95^th^ percentile. (b) Presence of CVC: type, duration of use, site. (c) Immobility: reduction in baseline mobility for >48 hours. (d) Infection: systemic or severe local infections, confirmed by blood or sputum culture, blood or respiratory viral assay, imaging tests or need for systemic antibiotics. (e) Pregnancy and postpartum. (f) Recent surgery: during the same hospitalization or in the two preceding weeks. (g) Trauma: in the two preceding weeks. (h) Family history: VTE in under 40-year-old family members, recurrent pregnancy loss with no apparent cause or severe thrombophilia in a first degree relative (antithrombin, protein C or protein S deficiency), prothrombin gene mutation or Factor V Leiden mutation in homozygosis or compound heterozygosis. (i) Prothrombotic drugs: combined oral contraceptives, corticoid or asparaginase. (j) ICU admission.

### Statistical analysis

A descriptive data analysis was conducted using absolute and relative frequencies for categorical variables. For the numerical variables, median, interquartile range (IQR), minimum and maximum values were used due to the non-normal distribution. The incidence of hospital-acquired thrombosis was calculated.

## Results

Over the 14-month study period, 18,038 children/adolescents were admitted to this institute and 45 cases of VTE were identified. Of these, four were excluded due to a diagnosis of chronic thrombosis and two were considered losses as the study interview could not be conducted because the patients died of severe sepsis less than 48 hours after the diagnosis of VTE. Therefore, 39 patients were included in the study.

The rate of hospital-acquired thrombosis was 19.9 cases per 10,000 pediatric hospitalizations, taking into consideration three patients who already had signs and symptoms of VTE at admission. Two of these were previously healthy children with a current infection (septic arthritis of the knee with DVT in the same limb and mastoiditis complicated with cerebral venous sinus thrombosis), while the third had complex congenital heart disease and polycythemia, with DVT in a lower limb.

The biological characteristics and comorbidities of the 39 cases of VTE are shown in Table 1. The median age was four months (range: 12 days-17 years). Most of the patients were male (53.8%) and mixed race (69.2%), while 48.7% presented with some thrombogenic comorbidity.

**Table 1:** Biological characteristics and comorbidities of the 39 children and adolescents with confirmed diagnoses of venous thromboembolism.

Characteristic	N	%
**Age group**		
0 to <30 days	10	25.6
30 days to <1 year	12	30.8
1 year to <5 years	4	10.3
5 to <12 years	7	17.9
12 to <18 years	6	15.4
**Sex**		
Male	21	53.8
Female	17	43.6
Undefined (ambiguous genitalia)	1	2.6
**Ethnicity**		
Mixed	27	69.2
White	10	25.6
Black	2	5.2
Asian/indigenous	0	0
**Thrombogenic comorbidities**		
Congenital heart disease	15	38.5
Cancer	4	10.3

A third of the patients (33.3%) had signs or symptoms of VTE with the others being diagnosed incidentally by imaging tests performed for other reasons. Table 2 shows the clinical characteristics of these patients. Thirty-five patients (89.7%) had CVCs implanted (with one patient having two CVCs implanted simultaneously): 32 CVCs were non-tunneled and four were peripherally inserted central catheters.

**Table 2:** Clinical characteristics of the 39 children and adolescents with a diagnosis of venous thromboembolism.

Characteristic	n	%
**Signs and symptoms** (n = 13)		
Edema	10	76.9
Hyperemia	5	38.5
Pain	1	7.7
Others^a^	3	23.1
**Implanted CVC** (n = 36)		
Site		
Subclavian vein	10	27.8
Brachiocephalic trunk	10	27.8
Jugular vein	7	19.4
Femoral vein	4	11.1
Axillary vein/basilic vein	1 each	5.6
Unrecorded (PICC)	3	8.3
Duration of CVC until diagnosis of VTE (days)		
Median, IQR and range (minimum-maximum)	10 (6-14); 2-180	
**Immobility**	32	82.1
**Infection** (n = 35)		
Systemic infection	22	62.9
Severe local infection	13	37.1
**Site of VTE** (n = 40^b^)		
Intracardiac	15	37.5
Cervical	8	20
Lower limb	6	15
Upper limb	3	7.5
Cerebral	2	5.0
Pulmonary embolism	1	2.5
Others^c^	5	12.5
**Days of hospitalization**		
Median, IQR and range (minimum-maximum)	19 (12-36);3-60	
**Time in ICU**	31	79.5
Days in ICU until diagnosis:		
Median, IQR and range (minimum-maximum)	18 (10-31); 2-59	
**Need for AMV**	20	51.3
Days on AMV until diagnosis:		
Median, IQR and range (minimum-maximum)	14 (6-23); 1-50	

VTE: venous thromboembolism; CVC: central venous catheter; PICC: peripherally inserted central catheter; ICU: intensive care unit; AMV: assisted mechanical ventilation, IQR: interquartile range.

a Two patients with signs and symptoms of cerebral venous sinus thrombosis (dizziness, headache, involuntary movements) and one with CVC malfunction.

b One patient had thrombosis diagnosed at two different sites in different exams.

c One in the superior vena cava, one in the inferior vena cava and three in the brachiocephalic trunk/subclavian vein.

The time between CVC placement and the radiological diagnosis of VTE was >7 days in 24 cases and >28 days in three cases. The longest duration of CVC use was six months. The frequency of infection was 89.7%. Of the 22 systemic infections recorded, blood cultures were positive in 18 cases with six being fungal infections (under 12-month-old patients with implanted CVCs), while four had signs of sepsis, although the cultures were negative (Table 2).

The diagnostic tests performed were Doppler ultrasonography (42.5%), echocardiogram (42.5%), CT with contrast (7.5%), CT angiography (2.5%) and magnetic resonance angiography (5.0%). One patient was affected by thrombosis at two different sites: intracardiac and the right subclavian vein. Intracardiac thrombosis was the principal diagnosis (37.5% of cases) and one patient had a diagnosis of PE (Table 2). Table 2 also shows the days of hospitalization, ICU stay and of AMV prior to the diagnosis of VTE.

There was a history of recurrent pregnancy loss of no apparent cause in the mother of one patient. There were no diagnosed cases of thrombophilia or thrombosis in any patient or family member. Three patients with a diagnosis of acute leukemia were using prothrombotic drugs; all three were taking corticoids (prednisone) and two were also taking asparaginase. None of the patients used oral contraceptives. Only one patient, a 14-year-old girl admitted to the obstetric ICU, was on anticoagulant prophylaxis following the institute's protocol for the prevention of thrombosis in adults.

Twelve patients had recently undergone surgery. The median time between surgery and diagnosis of VTE was 11.5 days (range: 1-33 days; interquartile range [IQR]: 7-16.5 days). One adolescent patient had a history of knee trauma that progressed to septic arthritis, with thrombosis in the same limb.

There were no cases of idiopathic VTE. Most patients had multiple risk factors for thrombosis, with ≥4 factors being identified in 74.6% of cases. The risk factors for VTE are described in Table 3. There were no cases of nephrotic syndrome, autoimmune or inflammatory disease, or obesity. The BMI could not be calculated for four of the 16 patients over two years old because their height was not recorded.

**Table 3:** Risk factors for venous thromboembolism in 39 children and adolescents.

Risk Factor	n	%
Presence of CVC	35	89.7
Infection	35	89.7
Immobility	32	82.1
Admitted to the ICU	31	79.5
Thrombogenic comorbidities	19	48.7
Recent surgery	12	30.8
Prothrombotic drugs	3	7.7
Trauma	1	2.6
Pregnancy/postpartum	1	2.6
Family history	1	2.6

CVC: Central venous catheter; ICU: Intensive care unit.

Thirty-seven patients (94.9%) received some form of treatment for thrombosis: enoxaparin in 94.6%, non-fractional heparin in 2.7% and thrombectomy in 2.7% of cases (failure to respond to enoxaparin, with thrombosis extending from the superior vena cava to the right atrium). Two patients received no treatment due to the high risk of bleeding. Four patients (10.3%) died; however, no death was directly related to the thrombosis. One was due to decompensation of congenital heart disease and the other three due to infection including sepsis.

## Discussion

This is the first study to evaluate the incidence and characteristics of pediatric cases of VTE in Brazil. A search of the principal databases (PubMed, LILACS, CAPES) using VTE-associated descriptors (venous thromboembolism, thrombosis) in pediatric patients (pediatrics, child, adolescent) revealed no similar studies conducted in this country. Thirty-nine children and adolescents diagnosed with VTE were analyzed, revealing an incidence of 19.9 cases of hospital-acquired VTE per 10,000 pediatric hospitalizations.

The incidence of hospital-acquired VTE in the present study was lower than recent North American reports.^5^^,^^22^ In a multicenter US study, the incidence of VTE per 10,000 pediatric hospitalizations increased from 34 in 2001 to 58 in 2007, reaching as high as 106 cases per 10,000 pediatric hospitalizations in 2019.^5^^,^^6^ Conversely, the rate found here is higher than that reported for Canada in the 1990s and for Pakistan in 2021 (5.3 and 5.9 per 10,000 pediatric hospital admissions, respectively).^1^^,^^17^

Slower progress in the medical interventions that should allow more patients with severe clinical conditions to survive, together with poorer professional training in recognizing pediatric VTE in developing countries could possibly explain the lower rate found in the present study.^17^ Furthermore, the relatively few adolescents, the possibility that suitable patients for the study were not identified, and the probable under-diagnosis of PE (signs and symptoms commonly confused with other more common diseases in the pediatric population) are other causes that may have contributed.

Although there was a predominance of VTE in under 12-month-old infants, similar to reports in the literature, the present study failed to find the classic bimodal distribution.^1^^,^^2^^,^^6^^,^^9^ The fact that there were relatively few adolescents in the sample was due to how the institute is organized, since, with the exception of certain sub-specialties, the cut-off age for general pediatric admission is 14 years. Conversely, the fact that newborn infants comprised a quarter of the sample reflects the institutional profile of this referral center for high-risk pregnancy and neonatal diseases.

Congenital heart disease was diagnosed in 60% of the newborns and gastroschisis in another 20%. The proportion of newborns varies greatly across studies (15-47%), with a tendency to be lower (9%) in developing countries.^2^^,^^5^^,^^6^^,^^17^ Some studies exclude the neonatal age group, mainly due to differences in data collection procedures, in risk factors and in VTE sites.^2^^,^^9^^,^^16^^,^^23^

Almost half the patients had some form of thrombogenic comorbidity. There is a lack of standardized reporting regarding what these comorbidities might be, particularly in the older studies.^1^^,^^5^^,^^16^ Nevertheless, considering the comorbidities reported in the present study and based on the ISTH recommendations (nephrotic syndrome, autoimmune and inflammatory disease, congenital heart disease, obesity) values reported in the literature range from 20% to 66%.^2^^,^^6^^,^^17^^,^^21^^,^^23^

According to the literature, 8-35% of pediatric patients with VTE have a diagnosis of cancer, which is in agreement with the present findings.^5^^,^^6^^,^^17^^,^^23^ Regarding congenital heart disease, the rate found here was higher than that previously reported (7.6-38%).^2^^,^^5^^,^^6^^,^^17^^,^^23^ The highest rates reported were from multicenter North American studies, with values similar to those reported here, probably because they also included referral centers in pediatric cardiology.^5^^,^^6^

Regarding clinically unsuspected VTE, data on its epidemiology, importance and outcome in the pediatric population remain sparse and poorly defined; however, it has been reported that over 90% of cases are associated with CVC use.^20^^,^^24^ Most studies have excluded cases of clinically unsuspected VTE or failed to differentiate them from symptomatic cases.^5^^,^^6^^,^^16^^,^^17^ When reported, clinically unsuspected VTE represents 15-35% of cases of pediatric VTE.^2^^,^^23^ Further data on this diagnosis is essential to ensure that children and adolescents are treated appropriately. The current recommendation is to individualize the indication of anticoagulants in these patients.^20^^,^^24^

Over half the patients (66.7%) in the present study were diagnosed incidentally and classified as having clinically unsuspected VTE. Of these, 96.1% had catheter-related thrombosis, with only one of these patients receiving no treatment for the thrombosis. No explanations were found for the different treatments given, which may reflect the fact that the decision was made by professionals other than a pediatric hematologist in 96.1% of cases (general pediatrician, neonatologist, cardiologist or pediatric intensive care specialist). The inclusion of many patients based on their anticoagulant use represents a bias that may also explain the high percentage of treatment, since the loss of untreated patients should have been greater than those treated.

Only one patient, admitted to the obstetric ICU, received anticoagulant prophylaxis, as established in the institute's protocol for adults. As in many pediatric units, there are no established protocols for thromboprophylaxis in the pediatric ICUs at this institute, with cases being individualized. There is no consensus in the current literature regarding prophylactic treatment for thrombosis in the pediatric population and risk scores are currently being developed to aid decision-making in as safe a manner as possible.^11^^,^^15^ Nevertheless, it remains unknown to what extent these scores could be extrapolated to populations from different countries or regions with different characteristics.

The presence of a CVC is well established as the single risk factor most associated with the development of VTE; however, in the present study the rate of CVC use was higher than that described in developed countries (55-80%).^13^^,^^23^ In underdeveloped countries where CVC use is less common, rates are even lower (32-43%), similar to the rate of 32% found in Canada in the 1990s.^1^^,^^16^^,^^17^ The probable explanation for this finding is the large number of critically ill patients evaluated in the present study, as supported by the cases of immobility, ICU admission and AMV use. Furthermore, a quarter of these patients were newborns (all of whom had implanted CVCs) and the association between CVC and VTE is known to be higher in this age group.^2^^,^^9^

Although there is not an established ideal CVC indwelling time, several studies chose seven days or 28 days as a cutoff point to increase the risk of VTE. This time is described as the most important risk factor for CVC-related VTE.^24^

The infection rate was much higher (89.7%) in the present study compared to rates of 23-46% in most other studies; these rates increase to 60% when only newborns are evaluated.^2^^,^^16^^,^^17^^,^^23^^,^^25^ There is a notable lack of consensus regarding the definition of ‘infection’ in studies.^2^^,^^16^^,^^17^^,^^23^^,^^26^ Here, the ISTH definition was used and the frequency of cases of systemic infection with positive blood cultures was 46.1%.^21^

The overall lethality of 10.3% found in the present study is in agreement with the current literature, with reported values of from 2-17%.^2^^,^^5^^,^^16^^,^^17^^,^^23^^,^^25^ Mortality tends to be higher in younger patients; three of the patients who died in this study were newborns.^25^

Despite the importance of this study, certain limitations must be taken into account. The sample consisted of only 39 patients at one single referral center that does not receive trauma victims. Furthermore, the age limit of 14 years for general pediatric admissions reduced the number of adolescents in the study. Nevertheless, an active search was conducted for cases of VTE over a 14-month period and the percentage of losses was small.

## Conclusions

These findings represent a starting point for the characterization of VTE in the pediatric population in Brazil. The presence of CVC and infection were the principal risk factors for VTE and were even more prevalent than described in the literature. Particular attention should also be given to the groups of newborns and children with heart disease. Understanding the situation of children and adolescents with VTE in different developing countries, and particularly in our own country, is the first step towards being able to identify cases of thrombosis at an early stage and identifying groups at risk. Consequently, scores and thromboprophylaxis strategies can be created. Carrying out multicenter studies in Brazil is crucial to understanding the situation in the Brazilian population.

## Conflicts of interest

None declared.
